# Statistical Analysis and Machine Learning Prediction of Disease Outcomes for COVID-19 and Pneumonia Patients

**DOI:** 10.3389/fcimb.2022.838749

**Published:** 2022-04-19

**Authors:** Yu Zhao, Rusen Zhang, Yi Zhong, Jingjing Wang, Zuquan Weng, Heng Luo, Cunrong Chen

**Affiliations:** ^1^College of Computer and Data Science, Fuzhou University, Fuzhou, China; ^2^Centre for Big Data Research in Burns and Trauma, Fuzhou University, Fuzhou, China; ^3^Department of Cardiovascular Medicine, Affiliated Fuzhou First Hospital of Fujian Medical University, Fuzhou, China; ^4^Department of Critical Care Medicine, Union Hospital of Fujian Medical University, Fuzhou, China; ^5^College of Biological Science and Engineering, Fuzhou University, Fuzhou, China; ^6^MetaNovas Biotech Inc., Foster City, CA, United States

**Keywords:** the coronavirus disease 2019, pneumonia, statistical analysis, machine learning, attention mechanism, clinical indicators

## Abstract

The Coronavirus Disease 2019 (COVID-19) has spread all over the world and impacted many people’s lives. The characteristics of COVID-19 and other types of pneumonia have both similarities and differences, which confused doctors initially to separate and understand them. Here we presented a retrospective analysis for both COVID-19 and other types of pneumonia by combining the COVID-19 clinical data, eICU and MIMIC-III databases. Machine learning models, including logistic regression, random forest, XGBoost and deep learning neural networks, were developed to predict the severity of COVID-19 infections as well as the mortality of pneumonia patients in intensive care units (ICU). Statistical analysis and feature interpretation, including the analysis of two-level attention mechanisms on both temporal and non-temporal features, were utilized to understand the associations between different clinical variables and disease outcomes. For the COVID-19 data, the XGBoost model obtained the best performance on the test set (AUROC = 1.000 and AUPRC = 0.833). On the MIMIC-III and eICU pneumonia datasets, our deep learning model (Bi-LSTM_Attn) was able to identify clinical variables associated with death of pneumonia patients (AUROC = 0.924 and AUPRC = 0.802 for 24-hour observation window and 12-hour prediction window). The results highlighted clinical indicators, such as the lymphocyte counts, that may help the doctors to predict the disease progression and outcomes for both COVID-19 and other types of pneumonia.

## 1 Highlights

1. In this study, multiple approaches including statistical analysis, machine learning and deep learning were utilized to understand the relationship between clinical variables and disease outcomes of COVID-19 and pneumonia.
2. The best models obtained good performance on the test sets when predicting severity of COVID-19 and the mortality of pneumonia patients in ICUs with AUROC larger than 0.9 and AUPRC greater than 0.8.
3. Feature importance and two-level attention mechanisms were utilized to interpret the models and highlight clinical variables associated with disease outcomes. Lymphocyte counts were found to be an important biomarker shared by COVID-19 and other types of pneumonia.

## 2 Introduction

In December 2019, an outbreak of highly infectious respiratory disease was reported in Wuhan, China, which was later identified as Coronavirus Disease 2019 (COVID-19) (Ward et al., 2020; Yamasaki et al., 2020). Ever since, COVID-19 has spread all over the world and impacted many people’s lives (Velavan and Meyer, 2020). In order to defeat COVID-19 pandemic, researchers developed machine learning models to help predict the outbreak so that the medical system can get ready and allocate resources ahead of time (Ardabili et al., 2020a; Ardabili et al., 2020b; Niazkar and Niazkar, 2020). Additionally, machine learning has also been utilized to help with the diagnosis and understand the clinical indicators of COVID-19 (Hussain et al., 2020; Li et al., 2020; Podder and Mondal, 2020; Alballa and Al-Turaiki, 2021; Podder et al., 2021). One of the major symptoms caused by COVID-19 is pneumonia, which is also a common infectious diseases threatening both human health and medical resources (Zimlichman et al., 2013). Patients with mild pneumonia only need outpatient treatment, while severe patients have to be hospitalized or even admitted to the intensive care unit (ICU) for rescue (Wei, 2020). Timely treatment for severe patients is significantly associated with reduced mortality (Attaway et al., 2021). Therefore, it is important to have early assess of the disease stage and make effective treatment plans (Watkins, 2020; González-Nóvoa et al., 2021).

In the early days, to classify the severity of respiratory and infectious diseases, the disease research societies have proposed different scoring standards (Liapikou et al., 2009). Among them, CURB-65, a combination of five endpoints, confusion, uremia, respiratory rate, blood pressure and age (65 years as a cutoff), is widely assessed in patients with community-acquired pneumonia (CAP) (Jones et al., 2011; Satici et al., 2020). For the COVID-19 patients, researchers also used CURB-65 to predict the risk of death (Nguyen et al., 2020). In addition to CURB-65, Zhang et al. (2019) developed a classification and regression tree (CART) scoring system by machine learning based on demographic data and clinical characteristics to evaluate the survival of CAP patients in ICUs. The results of Zhang et al. (2019) showed that the CART obtained better evaluation performance compared to CURB-65. Goodwin and Demner-Fushman, (2019)  further proposed a deep learning system, PRONTO, to predict the risk of patient death 1-2 days ahead. Based on extracorporeal membranous oxygenation (ECMO), Zhou et al. (2020) designed the pneumonia ECMO-eligible risk (PEER) system, which can predict the risk of death in a nomogram for both low-risk and high-risk pneumonia patients in the future. Yan et al. (2020) developed a machine learning model to propose a clinical decision rule composed of three biomarkers for COVID-19 patients, which can predict patient mortality 10 days in advance. Although many models have been developed to predict the disease outcomes, fewer studies focused on feature interpretability or clinical variable analysis across disease types. Even though some researchers compared differences between COVID-19 and regular pneumonia (Zhao et al., 2020), due to heterogenicity of different data sources, more analysis is helpful for a deeper understanding and better treatment approaches. Therefore, we compared and analyzed the clinical features of patients with COVID-19 and other types of pneumonia and assessed their performance of predicting disease outcomes.

In this study, a combination of machine learning models, including XGBoost and deep learning, was developed to predict the severity of COVID-19 and the patient mortality of other types of pneumonia in different time windows. Feature importance of both temporal and discrete clinical variables was analyzed to understand the association factors for different disease outcomes. Our goal is to provide early warnings for patients with dire outcomes so that doctors could have time to come up with appropriate monitoring and intervention procedures to prevent a worse situation.

## 3 Materials and Methods

### 3.1 The Overall Framework

**Figure 1:**
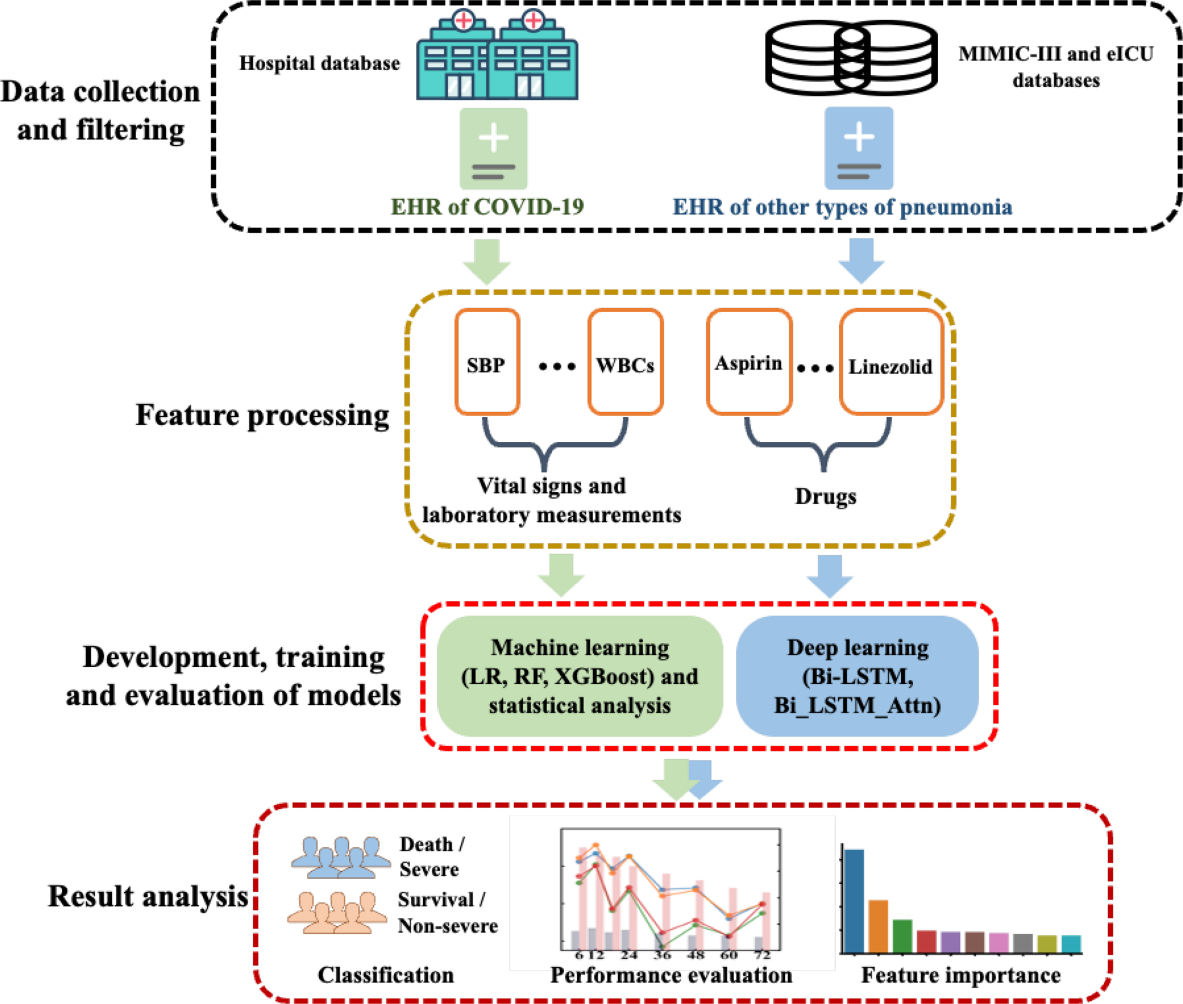
The overall workflow of this study. EHR, electronic health record; LR, logistic regression; RF, random forest; SBP, systolic blood pressure; WBCs, white blood cell counts.

The overall workflow of this study is summarized in **Figure 1**, which includes the following steps: (1) data collection of COVID-19 and other pneumonia data, (2) statistical analysis and development, training and evaluation of machine learning and deep learning models, and (3) result analysis including model evaluation and feature interpretation. The details were introduced in the following sections.

### 3.2 Collection and Analysis of COVID-19 Clinical Data

The demographic and clinical data of 80 COVID-19 patients were collected during the diagnosis and treatment of patients in Hubei province from February 1, 2020 to March 30, 2020, assisted by a medical team from Fujian Medical University Union Hospital. The study followed the principles of good clinical practice (GCP) and relevant national legal requirements with consent and approval from the patients’ families. It was also approved by the Ethics Committee of Fujian Medical University Union Hospital (No. 2021KY052).

Patients from this dataset were divided into severe COVID-19 (SC) and non-severe COVID-19 (non-SC) groups. Patients without any severity information were excluded. The missing clinical measurements were filled with average values.

To analyze the data, mean (along with standard deviation) or median (along with interquartile range (IQR)) values were calculated as numeric measurements. The Kolmogorov-Smirnov normality test was used to examine if the sample data were normally distributed. Additional statistical analysis methods including T-test, Kruskal Wallis rank test and Fisher’s exact test were utilized to summarize and analyze the clinical features of COVID-19 patients (Zhang et al., 2018). A *p* value less than 0.05 was considered statistically significant.

### 3.3 Collection and Processing of MIMIC-III and eICU Data

**Figure 2:**
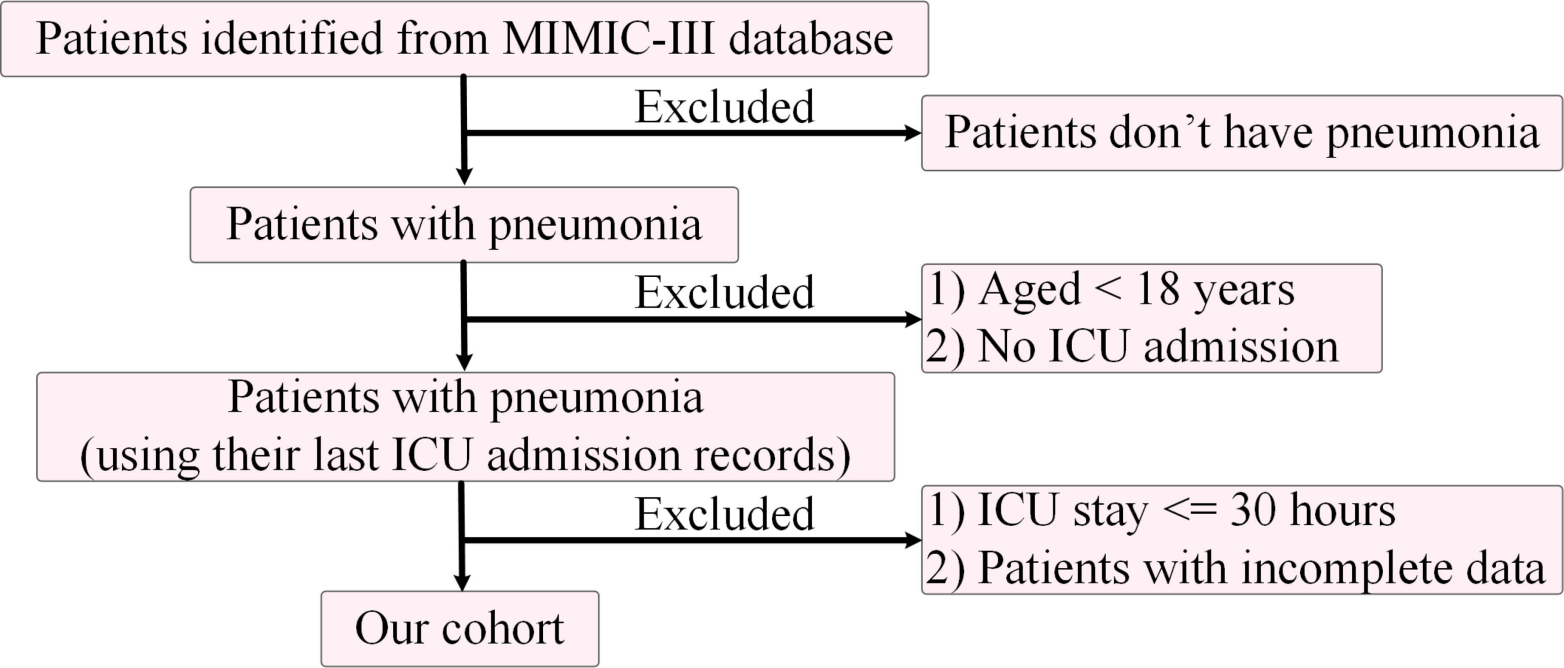
The flowchart of pneumonia patient selection from the MIMIC-III database.

The Medical Information Mart for Intensive Care (MIMIC-III) is a database that collected de-identified health records of over 50,000 patients who stayed in critical care units of Beth Israel Deaconess Medical Center between 2001 and 2012 (Johnson et al., 2016). Another database, eICU, is a combined clinical database from many ICUs in the United States. It contains clinical data of more than 200,000 hospitalized patients from 2014 to 2015 (Pollard et al., 2019). Since both the MIMIC-III and eICU data were harvested much earlier than the COVID-19 pandemic, it is safe to assume there were no COVID-19 patients in the records. The clinical data included demographics, vital signs, laboratory test results, diagnoses and other information of the patients. In this study, more than 3,000 patients with pneumonia by the International Classification of Diseases, Ninth Revision (ICD-9) codes of 481, 482 and 486 (**Table 1**) were identified in both MIMIC-III and eICU. Multiple filters were used to filter the patients (**Figure 2**). Adolescents and patients who did not stay in an ICU were excluded, and only patients with lengths of ICU stay no less than 30 hours were extracted.

**Table 1:** ICD-9 codes used in this study for pneumonia.

ICD-9 code	Description
481	Pneumococcal pneumonia [Streptococcus pneumoniae pneumonia]
482	Other bacterial pneumonia
486	Pneumonia, organism unspecified

**Figure 3:**
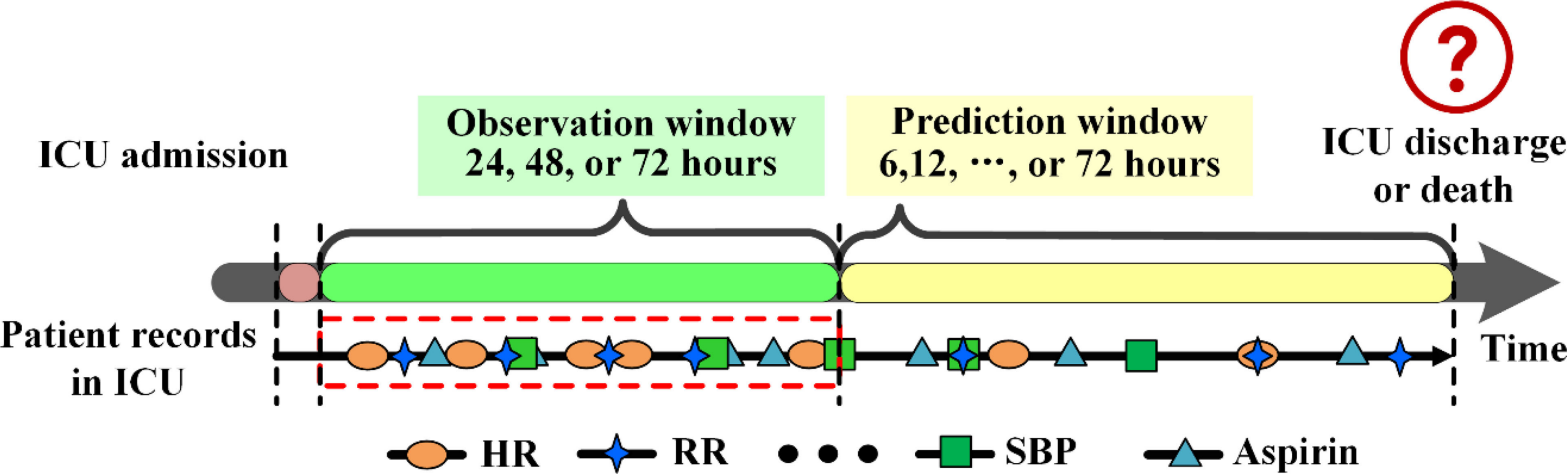
Data extraction framework for pneumonia patients in MIMIC-III. DBP, diastolic blood pressure; HR, heart rate; RR, respiratory rate; SBP, systolic blood pressure.

The pneumonia patients from MIMIC-III and eICU were grouped into two groups, survival or death, based on the records of ICU admission, discharge and death information. We referred Ng et al. (2016) for data extraction. Given an outcome of ICU discharge (survival) or death, the period right before the outcome was considered as the prediction window (6, 12, 24, 36, 48, 60 or 72 hours), and the time before the prediction window was used as the observation window (24, 48 or 72 hours). The clinical records in the observation window were used as input to develop models to predict outcomes (**Figure 3**). The abnormal values in the observation windows were deleted according to the criteria of Harutyunyan et al. (2019).

### 3.4 Model Development

#### 3.4.1 Traditional Machine Learning Models

We used a series of machine learning models in the following sections to predict disease outcomes based on the clinical features for both COVID-19 and other types of pneumonia.

##### 3.4.1.1 Logistic Regression (LR)

Logistic regression (Bouyer, 1991) is a generalized linear model used to solve classification problems. It is linear regression with a layer of the sigmoid function to map features towards labels. By introducing regularization, it can reduce the influence of multicollinearity. Given *n* data samples with *m* features as *x_j_* (*j*∈ (1,2, … ,*m*)), the predicted probability $\hat{y_{i}}$ of sample *i* can be calculated as follows:


$${\hat{y}}_{i}=\frac{1}{1+e^{-\theta^{T}x^{\left(i\right)}}}$$

$$\theta^{T}X^{\left(i\right)}=\;\theta_{0}+\sum_{j=1}^{m}\theta_{j}x_{j}^{\left(i\right)}$$

where *θ* represents coefficients.

##### 3.4.1.2 Random Forest (RF)

Random forest (Biau and Scornet, 2016) is composed of multiple decision trees (classified regression trees, or CARTs). The model randomly drafts *N* training subsets M = {*M*_1_, *M*_2_,…,*M_n_*} based on bootstraps. The probability *P* of each sample not being drawn is calculated as follows:


$$P\;={\left(1-\frac{1}{N}\right)}^{N}$$

The *N* decision trees T = {*T*_1_, *T*_2_,…,*T_n_*} are developed based on their corresponding training subsets. The Gini index is used for CARTs to select tree nodes. Gini index is calculated as follows:


$$G\left(M\right)=\sum_{k=1}^{K}p_{k}\left(1-p_{k}\right)=1-\sum_{k=1}^{K}p_{k}^{2}$$

Here *M* is the independent training subset, and *p_k_* indicates the probability that the sample belongs to the *k*-th category.

##### 3.4.1.3 XGBoost

XGBoost (Chen et al., 2015) is a gradient lifting tree model composed of multiple CARTs. The algorithm divides samples into two branches according to the feature thresholds. After multiple groupings, the end of each CART (leaf node) contains samples with the same label. Given *n* samples with *m* features as *x_i_* (*i*∈ (1,2, … , *m*)), the predicted probability ${\hat{y}}_{i}$ of sample *i* can be calculated as follows:


$${\hat{y}}_{i}=\sum_{K=1}^{K}f_{k}\left(x_{i}\right),f_{k}\in F$$

where *f_k_* is the prediction score of a single decision tree, and ϝ is the space of all trees.

In order to get the optimal solution, the following loss function with regularization is optimized:


$$ℒ\left(\phi \right)=\sum_{i}l\left({\hat{y}}_{i},y_{i}\right)+\sum_{k}\Omega \left(f_{k}\right)$$

$$\Omega \left(f\right)=\gamma T+\frac{1}{2}\lambda {\left\|\omega_{i}\right\|}^{2}$$

Where ℒ is the loss function, *Ω* is the penalty term, *T* is the number of leaves, *ω_i_* is the score of leaf node *i*, and *γ* and *λ* are the coefficient parameters.

##### 3.4.1.4 Data Processing

For the COVID-19 data, the latest clinical features measured during the hospitalization of the COVID-19 patients were used to train the models to classify the outcomes of severe COVID-19 (SC) and non-severe COVID-19 (non-SC).

For the MIMIC-III and eICU data, the distribution parameters (maximum, minimum, median, mean and standard deviation) of vital signs and laboratory measurements in the observation window, as well as the drug prescriptions were prepared as features. For the oxygenation indexes (SpO2 and SaO2), the maximum values were not used since most of them were close to 100%. The models were trained to predict the outcome of survival for each patient.

For logistic regression, both L1 and L2 regularizations were applied. The top 10 most important features in XGBoost were analyzed to understand the clinical indicators for disease outcomes.

#### 3.4.2 Deep Learning Models

##### 3.4.2.1 Model Design

Since the MIMIC-III and eICU data contain temporal clinical features, two time-series deep learning models, including bi-directional long short-term memory (LSTM) models without and with attention (namely Bi-LSTM and Bi-LSTM_Attn, respectively), were developed to predict survival of the pneumonia patients. However, due to the limited samples and discrete features of COVID-19 data, we were not able to apply these models and had to rely on the traditional models.

For MIMIC-III and eICU data, the clinical features include vital signs, laboratory measurements and drug usages (**Supplementary Table S1**). The clinical data of one-hour intervals from the observation windows were extracted from both databases. If there were multiple measurements within a given hour, their mean values were taken as features. If no data within the one-hour internal were observed, the mean values of the entire temporal series were used.

**Figure 4:**
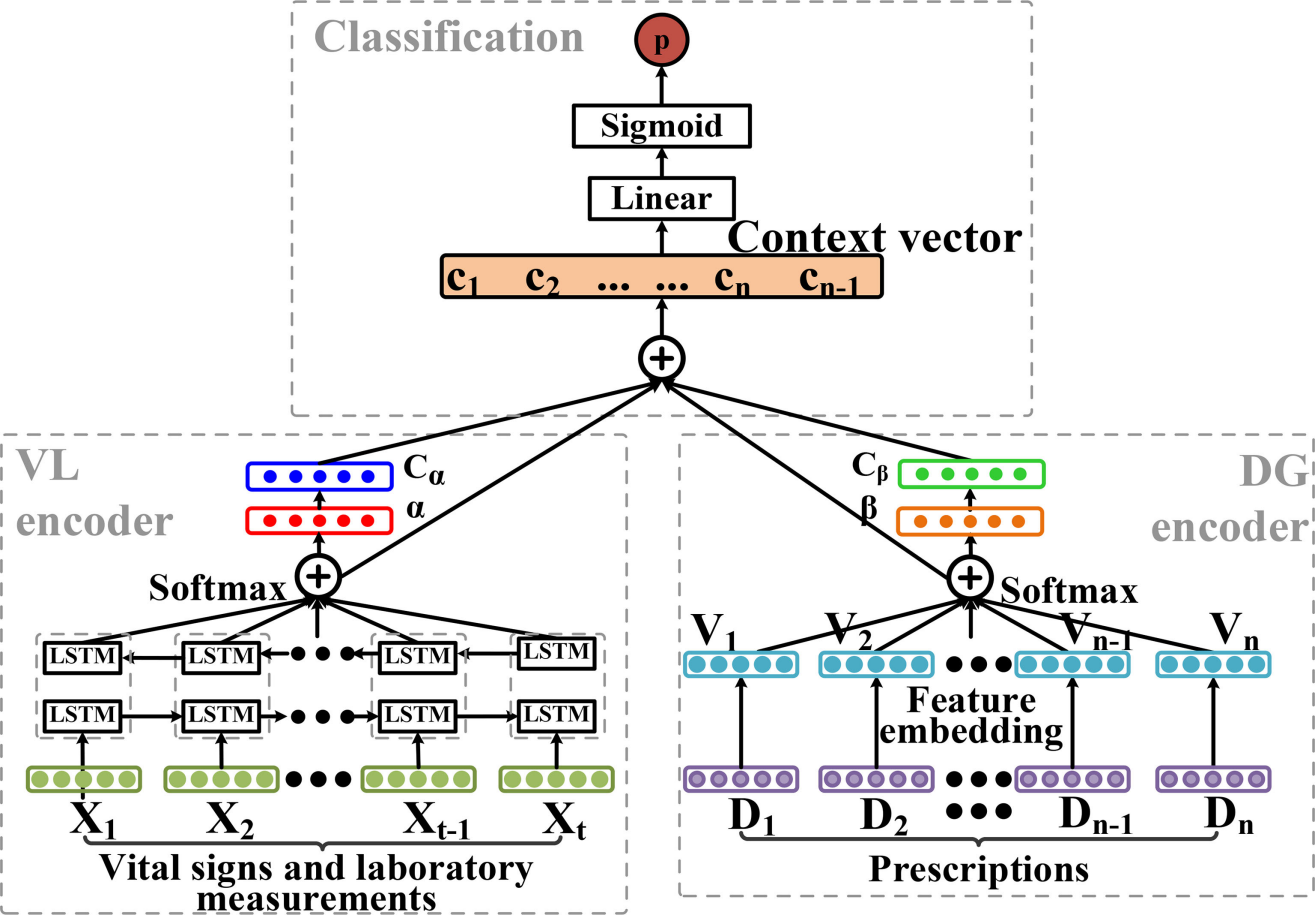
The deep learning model architecture of bi-directional long short-term memory (LSTM) with attention (Bi-LSTM_Attn). It consists of three parts: vital sign and laboratory measurement (VL) encoder, drug (DG) encoder and the classification module.

The architecture of the deep learning model (**Figure 4**) was developed based on Zhang et al. (Kaji et al., 2019) and the RETAIN (Choi et al., 2016) model. Taking the bi-directional long short-term memory (LSTM) model with attention (Bi-LSTM_Attn) as an example, the hourly data of vital signs and laboratory measurements in the observation windows were used as the inputs. Self-attention mechanisms were utilized to calculate the attention weights of both time-series clinical data and the discrete drug prescription features. Then both sides were concatenated by the context vector and a final sigmoid function was used to make a classification prediction. The model included three parts: 1) the vital sign and laboratory measurement (VL) encoder, 2) the drug (DG) encoder and 3) the classification module, which were introduced in the sections below.

The LSTM model without the attention layers (Bi-LSTM) had a similar but simpler architecture. Since no attention module was presented, the outputs from the head and tail LSTM modules were merged with the drug feature vectors and sent to the linear layers with the sigmoid function for classification.

##### 3.4.2.2 The VL Encoder

To obtain the embeddings of vital signs and laboratory measurements, we used the bidirectional LSTM (Bi-LSTM) networks, which combined both the forward and backward information within a sequence. Given a temporal feature vector at a specific time (t), $X_{i}^{t}\left(i\in \left\{1,2,3,\ldots ,N\right\},t\in \left\{1,2,3,\ldots ,T\right\}\right)$ of patient *i*, the embedding of the input vector $X_{i}^{\left(t\right)}$ was calculated by the Bi-LSTM model as follows:


$$h_{i}^{\left(t\right)}=Bi-LSTM\left(h_{i}^{\left(t-1\right)},X_{i}^{\left(t\right)}\right)$$

The bidirectional output vector $h_{i}^{\left(t\right)}$ was defined as $h_{i}^{\left(t\right)}=\left[{\vec{h}}_{i}^{\left(t\right)},{\overset{\leftarrow }{h}}_{i}^{\left(t\right)}\right]$ . Since $h_{i}^{\left(t\right)}$ contained the sequential information around $X_{i}^{\left(t\right)}$ , in order to let the model know the importance of each time point in the observation window, the attention weight of each moment was calculated by self-attention mechanism. The temporal context vector *c_α_* was calculated as follows:


$$\alpha_{i}^{\left(t\right)}=Softmax\left(W_{\alpha }^{T}h_{i}^{\left(t\right)}+b_{\alpha }\right)$$

$$c_{\alpha }=\sum_{i=1}^{T}\alpha_{i}^{\left(t\right)}h_{i}^{\left(t\right)}$$

Where $W_{\alpha }^{T}$ and *b_α_* are learnable parameters and $\alpha_{i}^{\left(t\right)}$ is the attention weight of each time point.

##### 3.4.2.3 The DG Encoder

Given a list of drug prescriptions for each patient as *D_i_* = [*d*_0_, *d*_1_, *d*_2_, …,*d_M_*] (*d_i_* ∈{0, 1}, *i* ∈{1, 2, 3, … , *M*}), multi-layer artificial neural networks were used to get the embedding vector of the prescription *v_i_* as *v_i_* = [*v_0_*,*v_1_*,*v_2_*,…,*v_M_*] follows:


$$V_{i}=MLP\left(D_{i}\right)$$

Here, the Rectified Linear Unit (ReLU) was utilized as the activation function. To help the model understand the importance of the drug prescription features, MLP layers were used to calculate attention weight *β_i_* for the embedding feature vector *V_i_* of each drug prescription. Then a sum of dot product of all the embedding vectors *V_i_* and attention vectors *β_i_* were calculated as follows:


$$\beta_{i}=Softmax\left(W_{\beta }^{T}V_{i}+b_{\beta }\right)$$

$$c_{\beta }=\sum_{i=1}^{T}\beta_{i}⊙V_{i}$$

Where $W_{\beta }^{T}$ and *b_β_* are learnable parameters, ⊙ is the element-wise multiplication and *c_β_* is the drug context vector.

Because the sample data we selected is unbalanced, so we use the class-balanced (CB) loss (Cui et al., 2019) to calculate the classification loss as follows:


$$Loss\left(p,y\right)=-\frac{1}{N}\sum_{i}^{N}\left(y_{i}log{\hat{y}}_{i}+\left(1-y_{i}\right)log\left(1-{\hat{y}}_{i}\right)\right)$$

$$Bloss\left(p,y\right)=-\frac{1}{E_{n_{y}}}Loss\left(p,y\right)=\frac{1-\beta }{1-\beta^{n_{y}}}Loss\left(p,y\right)$$

where N is total sample, *β* ∈[0,1) is a hyperparameter and *n_y_* is the number of *y* labels in the sample.

#### 3.4.3 Model Prediction and Evaluation

Since the COVID-19 dataset is a relatively small dataset, the COVID-19 patients were randomly divided into training (including validation) and test sets using 4:1 ratio in order to have enough patients in the test set. For the MIMIC-III and eICU data, pneumonia patients were randomly divided using 8:1:1 ratio into the training, validation and test sets. The training and validation sets were combined for 5-fold cross-validations during the development of traditional machine learning models. For deep learning models, the training set was used to train the models; the validation set was utilized to learn and optimize the hyperparameters and the test set was used to evaluate the models. The deep learning models contained a single-layer Bi-LSTM with 256 hidden layers to generate embeddings for temporal features, and two MLP layers (128 and 103 neurons) to generate embeddings for the drug prescription features. Both lists of embeddings were merged and sent to a linear layer of 128 neurons with a sigmoid activation function as the classification module. The dropout rate of 0.5 was used before the linear layer. During training, the Adam optimizer was used with a learning rate of 0.001, the batch size was set to 32, and the models were trained by 150 epochs. The area under the receiver operating characteristic curve (AUROC) and area under the precision-recall curve (AUPRC) were calculated to evaluate the model performance. Additional details regarding the evaluation metrics were attached in **Supplementary Materials**.

## 4 Results

### 4.1 Statistical Analysis of the COVID-19 Data

The statistical analysis for the COVID-19 data was shown in **Table 2**. Of the 80 COVID-19 patients, male accounted for 47.50%. The average age was 64.21 ± 20 years with Kolmogorov-Smirnov normality test *p* > 0.05, indicating a normal distribution. Cough (71.25%) and fever (63.75%) were the most frequent symptoms, while a few patients had other symptoms, such as shortness of breath (17.50%), diarrhea (5.00%) and chest pain (3.75%). Some patients had other diseases, including diabetes (23.75%), heart disease (5.00%), respiratory system disease (5.00%), other symptoms (28.75%). The median of eastern cooperative oncology group (ECOG) score and acute physiology and chronic health evaluation II (APACHE II) score were 1.00 [IQR, 1.00-2.00] and 5.00 [IQR, 3.00-7.25]. Among the drugs used in the treatment of COVID-19, arbidol was the most common one (83.75%), followed by oseltamivir (20.00%) and ribavirin (4.00%). For blood test results, the medians of albumin, lymphocyte counts and prothrombin time (PT) were 36.80 g/L [IQR, 33.30-40.65], 1.39×10^9^/L [IQR, 0.97-1.72] and 13.40 seconds (s) [IQR, 13.00-14.25], respectively.

**Table 2:** Statistics of the clinical variables for COVID-19 data.

Variables	Overall( n = 80)	SC groups (n = 13)	Non-SC groups (n = 67)	P value
**Demographic characteristics**				
Age, mean (SD) (years)	64.21 (13.20)	73.15 (13.64)	62.48 (12.48)	0.007
Gender, male, n (%)	38 (47.50%)	8 (61.54%)	30 (44.78%)	0.366
**Prognostic scoring system, median (IQR)**				
APACHE II	5.00 (3.00-7.25)	12.00 (11.00-16.00)	5.00 (3.00-6.00)	<0.001
ECOG	1.00 (1.00-2.00)	2.00 (1.75-3.00)	1.00 (1.00-1.00)	<0.001
**Symptoms on onset, n (%)**				
Chest pain	3 (3.75%)	1 (7.69%)	2 (2.99%)	0.417
Cough	57 (71.25%)	7 (53.85%)	50 (74.63%)	0.180
Diarrhea	4 (5.00%)	2 (15.38%)	2 (2.99%)	0.122
Fever	51 (63.75%)	7 (53.85%)	44 (65.67%)	0.531
Shortness of breath	14 (17.50%)	3 (23.08%)	11 (16.42%)	0.690
Other symptoms	23 (28.75%)	3 (23.08%)	20 (29.85%)	0.747
**Drug, n (%)**				
Arbidol	67 (83.75%)	10 (76.92%)	57 (85.07%)	0.435
Ribavirin	32 (40.00%)	6 (46.15%)	26 (38.81%)	0.759
Oseltamivir	16 (20.00%)	2 (15.38%)	14 (20.90%)	1.000
Other drugs	38 (47.50%)	5 (38.46%)	33 (49.25%)	0.554
**Comorbidities, n (%)**				
Diabetes	19 (23.75%)	3 (23.08%)	16 (23.88%)	1.000
Heart disease	4 (5.00%)	1 (7.69%)	3 (4.48%)	0.448
Hypertension	25 (31.25%)	4 (30.77%)	21 (31.34%)	1.000
Respiratory system disease	4 (5.00%)	2 (15.38%)	2 (2.99%)	0.086
Other diseases	12 (15.00%)	3 (23.08%)	9 (13.43%)	0.390
**Laboratory tests (last measurements)**				
Albumin, median (IQR) (g/L)	36.80 (33.30-40.65)	33.00 (30.30-35.50)	38.60 (34.55-41.25)	<0.001
ALT, median (IQR) (U/L)	25.50 (18.00-53.00)	20.00 (14.00-56.00)	26.00 (19.50-51.00)	0.389
APTT, median (IQR) (s)	37.20 (34.60-40.40)	38.80 (36.25-41.95)	36.50 (34.35-40.12)	0.088
AST, median (IQR) (U/L)	25.00 (20.00-35.50)	22.00 (16.00-34.00)	25.00 (20.00-36.00)	0.331
BUN, median (IQR) (mmol/L)	4.30 (3.50-5.45)	8.10 (4.75-10.91)	4.10 (3.50-5.00)	0.005
CD3 T, median (IQR)	76.46 (65.83-83.91)	60.82 (44.78-71.79)	77.85 (67.19-84.56)	0.027
CD4 T, mean (SD)	45.46 (14.10)	42.80 (14.90)	45.77 (14.10)	0.601
CD8 T, mean (SD)	23.87 (10.12)	19.56 (8.31)	24.37 (10.25)	0.237
CK, median (IQR) (IU/L)	61.00 (38.00-88.00)	99.50 (47.00-210.50)	61.00 (38.00-86.00)	0.277
Creatinine, median (IQR) (µmol/L)	76.00 (67.00-89.25)	87.00 (73.00-146.70)	74.00 (67.00-89.00)	0.130
CRP, median (IQR) (mg/L)	3.74 (3.14-18.10)	25.40 (11.97-95.97)	3.37 (2.96-13.45)	0.024
DB, median (IQR) (mg/L)	3.75 (2.70-5.20)	5.30 (4.10-9.60)	3.60 (2.65-4.65)	0.021
Fibrinogen, median (IQR) (g/L)	4.26 (3.29-5.26)	5.01 (3.83-6.21)	4.05 (3.23-5.05)	0.137
Glucose, median (IQR) (mmol/L)	5.59 (5.05-6.54)	6.79 (6.30-7.10)	5.52 (5.04-6.48)	0.089
Hemoglobin, mean (SD) (g/L)	117.99 (16.54)	105.69 (18.88)	120.37 (15.07)	0.003
LDH, median (IQR) (U/L)	187.50 (160.00-222.50)	201.00 (160.00-239.00)	185.00 (160.00-221.00)	0.643
Lymphocyte counts, median (IQR) (10^9^/L)	1.39 (0.97-1.72)	0.68 (0.43-0.84)	1.48 (1.20-1.77)	<0.001
Monocyte counts, median (IQR) (10^9^/L)	0.50 (0.35-0.69)	0.43 (0.31-0.70)	0.51 (0.35-0.68)	0.588
Neutrophil counts, median (IQR) (10^9^/L)	3.48 (2.40-4.90)	6.46 (2.76-11.37)	3.40 (2.38-4.46)	0.078
Platelet counts, mean (SD) (10^9^/L)	179.28 (62.48)	166.85 (89.37)	181.69 (56.43)	0.437
Procalcitonin, median (IQR) (µg/L)	0.13 (0.07-0.13)	0.13 (0.13-0.21)	0.13 (0.06-0.13)	0.021
PT, median (IQR) (s)	13.40 (13.00-14.25)	14.60 (14.50-15.65)	13.40 (12.97-14.00)	<0.001
TB, median (IQR) (mg/L)	10.95(9.07, 15.53)	15.00(9.70-23.50)	10.60 (8.95-15.00)	0.142
Total protein, median (IQR) (gl-1)	64.90 (60.98-68.90)	58.00 (54.40-63.00)	65.90 (62.45-69.65)	0.007
Troponin, median (IQR)	3.20 (1.65-6.45)	11.20 (8.65-21.70)	2.70 (1.58-5.65)	0.001
TT, median (IQR) (s)	18.00 (17.05-19.00)	17.70 (15.95-19.10)	18.00 (17.10-18.95)	0.441
Uric acid, median (IQR) (mg/L)	267.00 (215.00-326.50)	228.00 (215.00-279.00)	271.00 (215.50-327.50)	0.449
WBCs, median (IQR) (10^9^/L)	5.74 (4.23-7.30)	7.62 (3.48-12.18)	5.53 (4.48-6.87)	0.351

ALT, alanine aminotransferase; APACHE II, acute physiology and chronic health evaluation II; APTT, activated partial thromboplastin time; AST, aspartate aminotransferase; BUN, blood urea nitrogen; CK, Creatine kinase; CRP, C-reactive protein; DB, Direct bilirubin; ECOG, eastern cooperative oncology group; IQR, interquartile range; LDH, lactate dehydrogenase; non-SC, non-severe COVID-19; PT, prothrombin time; SC, severe COVID-19; TB, total bilirubin; TT, thrombin time; WBCs, white blood cell counts. For variables with normal distributions (Kolmogorov-Smirnov normality test p > 0.05), the mean and standard deviation (SD, in parentheses) values were given, and t-test was used to compare the severe COVID-19 (SC) group and the non-severe COVID-19 (non-SC) group. For variables with non-normal distributions, the median and interquartile range (IQR, in parentheses) were provided, and Kruskal Wallis rank test was used to compare SC vs non-SC groups. For categorical variables, the count and percentage (in parentheses) values were shown, and Fisher’s exact test was utilized to calculate the p values.

Of the 80 patients with COVID-19, 13 were severe COVID-19 (SC) patients and 67 were non-severe COVID-19 (non-SC) patients. The severe patients had an average age of 73.15 ± 13.64 years, while non-severe patients had a younger average age of 62.48 ± 12.48 years (t-test *p* < 0.05). Here, age may be a factor that is associated with the severeness of COVID-19. In addition, the prognosis scores, including ECOG scores and APACHE II scores of severe patients, were higher than those of the non-severe patients both with Kruskal Wallis rank test *p* < 0.001, as larger prognosis scores indicate more severe conditions. For blood test results, albumin and lymphocyte counts were lower in severe patients than non-severe patients, while the prothrombin time (PT) was longer in severe COVID-19 patients than non-severe COVID-19 patients (all Kruskal Wallis rank *p* values < 0.001).

### 4.2 Model Performance and Interpretation for the COVID-19 Data

**Figure 5:**
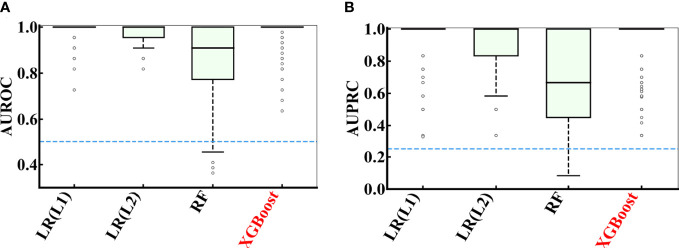
Performance comparison of multiple models during 100 rounds of 5-fold cross-validations on the COVID-19 data. **(A)** AUROC and **(B)** AUPRC curve showing the performance of the classifier using different model using cross-validation on the COVID-19 data.

**Figure 5** showed the distribution of AUROC and AUPRC values of different models after 100 rounds of 5-fold cross-validations on the COVID-19 data. The hyperparameters of the models were attached in **Supplementary Table S2**. All AUROC values of logistic regression with L1 regularization (LR(L1)), logistic regression with L2 regularization (LR(L2)) and XGBoost models in predicting the severity of patients with COVID-19 were greater than 0.5, and the AUPRC values of LR(L1), LR(L2) and XGBoost were all greater than 0.25. While random forest (RF) had slightly worse performance, LR(L1) (AUROC = 0.986 ± 0.033 and AUPRC = 0.948 ± 0.121), LR(L2) (AUROC = 0.983 ± 0.032 and AUPRC = 0.931 ± 0.135) and XGBoost (AUROC = 0.979 ± 0.048 and AUPRC = 0.927 ± 0.154) were more effective to predict the severity of COVID-19 patients.

**Figure 6:**
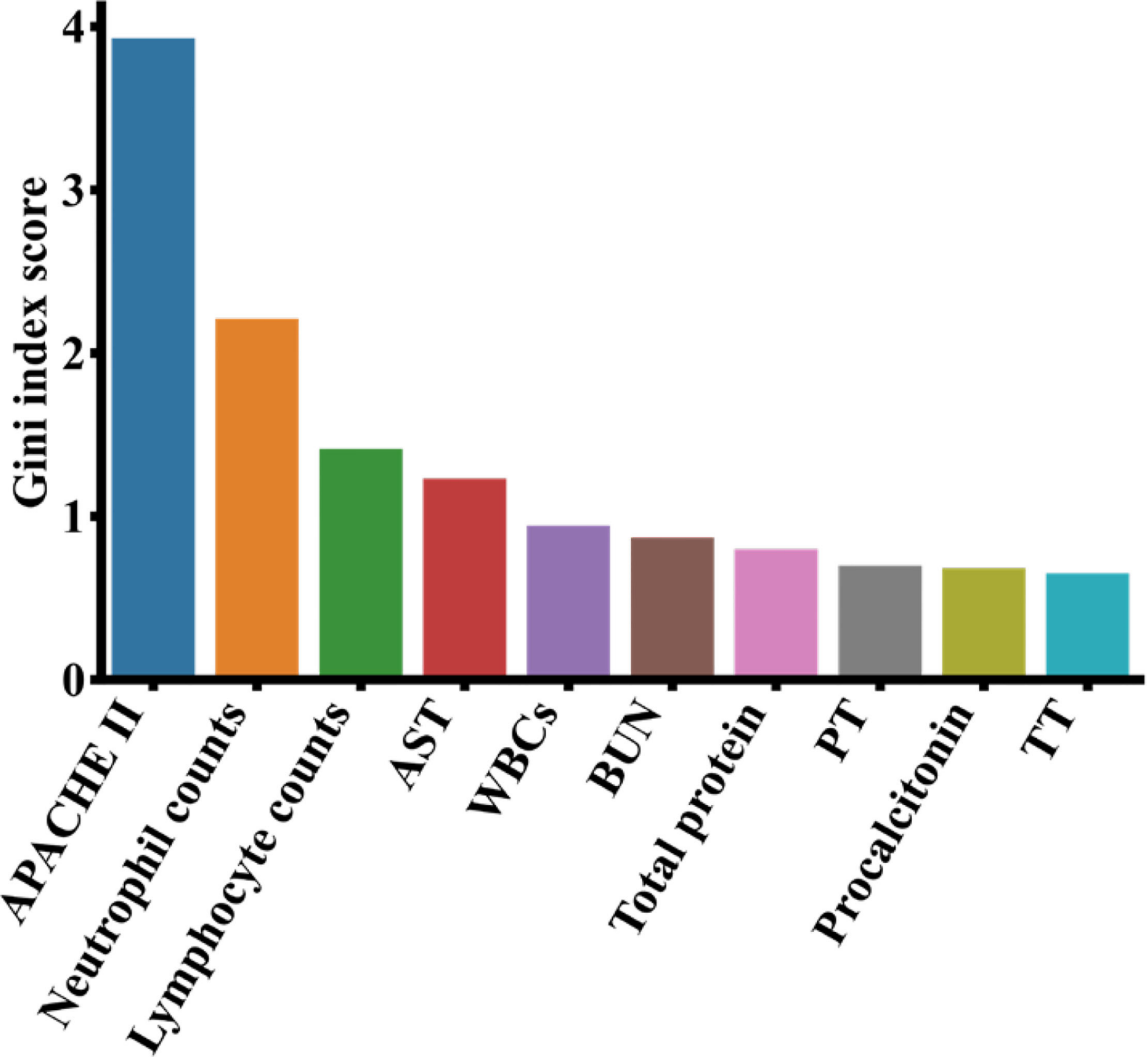
Top 10 most important features from the XGBoost model on the COVID-19 data. APACHE II, acute physiology and chronic health evaluation I; AST, aspartate aminotransferase; BUN, blood urea nitrogen; PT, prothrombin time; WBC, white blood cell count; TT, thrombin time.

For the XGBoost model, we calculated the contributions of each clinical variables towards the severity of COVID-19 by information gain, and showed the top 10 most important features in **Figure 6**. Results indicate that the APACHE II has the largest importance, followed by neutrophil counts, lymphocyte counts and AST (aspartate aminotransferase). In order to get an interpretable decision tree, we set the parameter n_estimators to 1 in XGBoost and fed the top 10 features into XGBoost one by one. Finally, we got the best decision tree from the APACHE II feature (AUROC = 1.00, AUPRC = 0.833) to predict the severity of COVID-19 patients (**Supplementary Figure S1**). Additionally, the top 10 most important features from the LR(L1), LR(L2) and RF models were included in **Supplementary Table S3**, which were found similar to the top features from the XGBoost model.

### 4.3 Model Performance and Interpretation of the MIMIC-III and eICU Data

**Figure 7:**
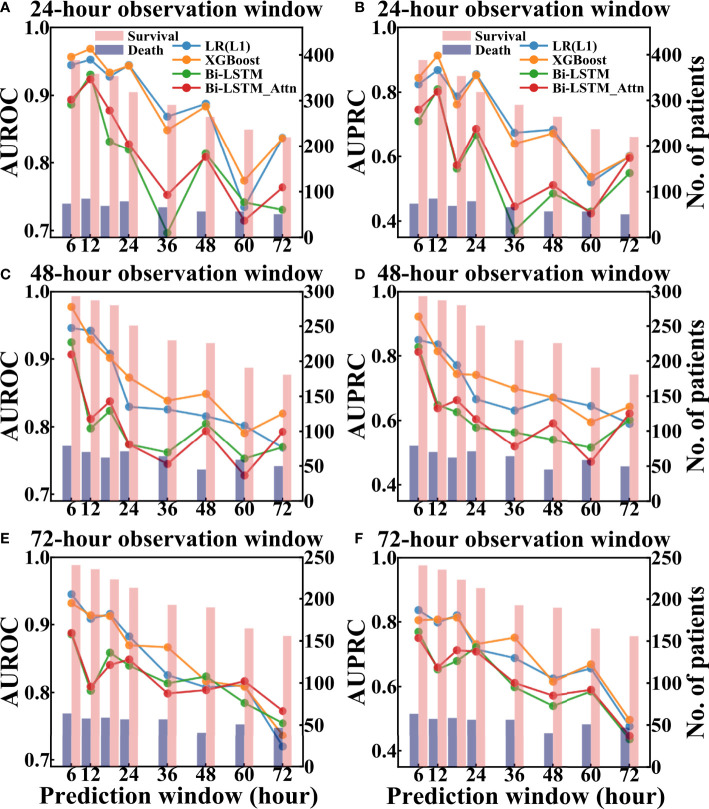
Performance of different models to predict pneumonia patient survival using different prediction and observation windows based on the test set of MIMIC-III. The left column showed AUROC values and the right column showed AUPRC values. **(A, B)** are AUROC and AUPRC of different models under different 24-hour observation windows and different prediction windows, respectively. **(C, D)** are AUROC and AUPRC of different models under different 48-hour observation windows and different prediction windows, respectively. **(E, F)** are AUROC and AUPRC of different models under different 72-hour observation windows and different prediction windows, respectively.

For MIMIC-III data, the performance of models was dependent on the selection of the observation and prediction windows (**Figure 7**). From the data point of view, shorter prediction window or observation window corresponded to more patients in the test set, especially the survival patients. For model performance, when the observation window was fixed, the performance of all models generally decreased as the length of the prediction window increased. This makes sense since longer prediction window presents more challenges for the models to predict the future.

The hyperparameters of LR(L1) and XGBoost models were attached in **Supplementary Table S4** and 10-fold and 15-fold cross-validation results of these models were included in **Supplementary Table S5**. Comparing across different models, the AUROC values of the traditional machine learning models (LR(L1) and XGBoost) were higher than the deep learning models when both the observation and prediction windows were within 48 hours. However, when both the observation and prediction window were large (no less than 60 hours), the Bi-LSTM_Attn model outperformed others in terms of AUROC [**Figure 7**(e)]. Since the deep learning models were designed to learn and infer based on temporal features, it was expected to obtain a better performance when time windows were longer.

**Figure 8:**
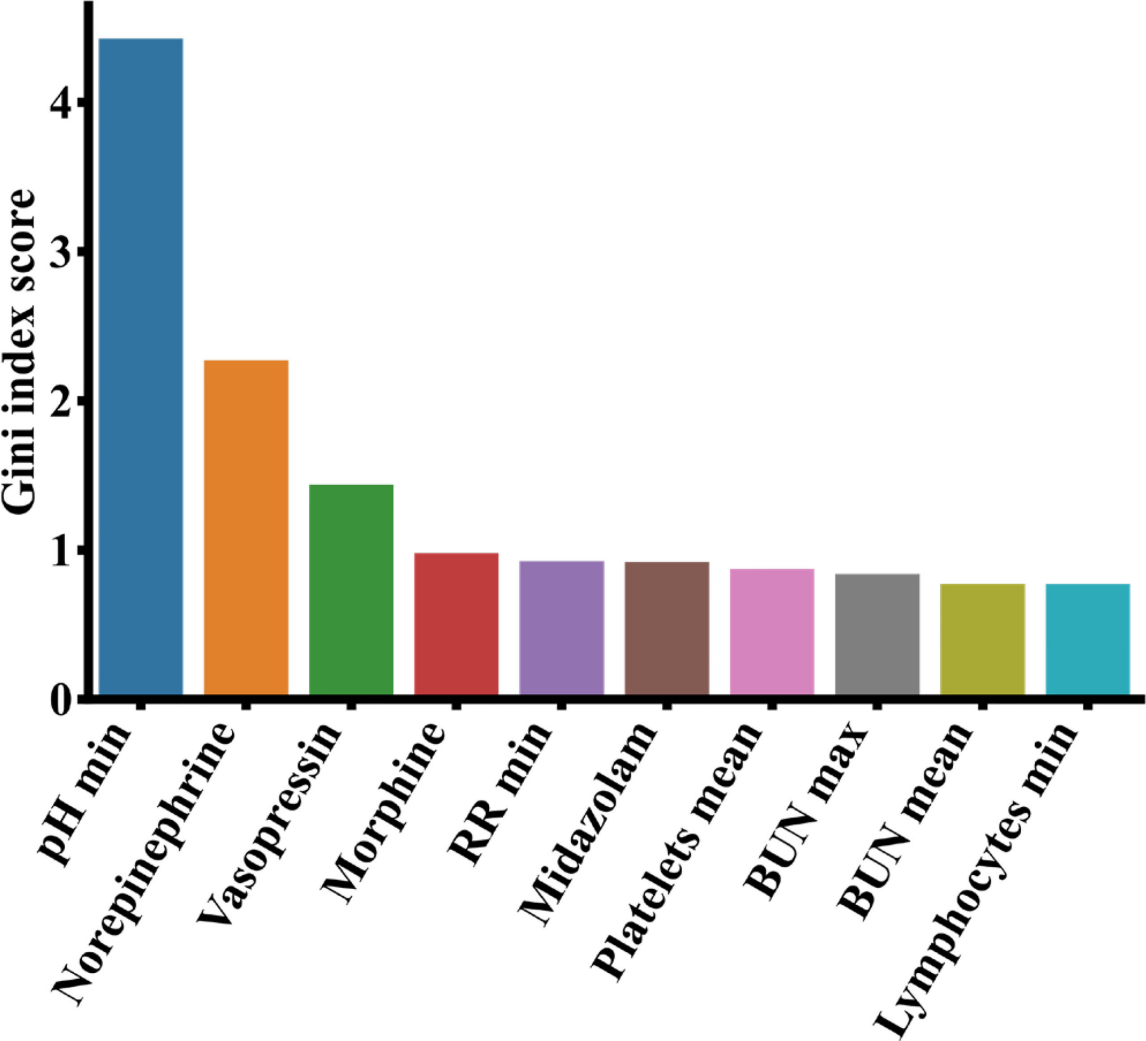
Top 10 most important features from the MIMIC-III data based on analysis of the XGBoost model. BUN, blood urea nitrogen; RR, respiratory rate.

Like before, we calculated the feature importance from the XGBoost model *via* information gain and showed the top 10 most important features in **Figure 8**. Among the 10 features, norepinephrine, vasopressin, morphine and midazolam were known drugs for the treatment of patients with pneumonia. Additionally, we found that the minimum respiratory rates (RR) value, the minimum pH, the average platelet count, the maximum and average blood urea nitrogen (BUN), and the lymphocytes minimum were important clinical features affecting the patient survival with pneumonia in ICUs.

For eICU data, the performance evaluation of the deep learning models (Bi-LSTM and Bi-LSTM_Attn) under different time windows was shown in **Supplementary Figure S2**. We found that for the eICU data, the best time window is consistent with MIMIC-III data for predicting the mortality of pneumonia patients during ICU stay (AUROC = 0.856 and AUPRC = 0.566), which is a 24-hour observation window and a 12-hour prediction window.

### 4.4 Visualization of the Attention Mechanism in the Deep Learning Model for Predicting MIMIC-III Pneumonia

**Figure 9:**
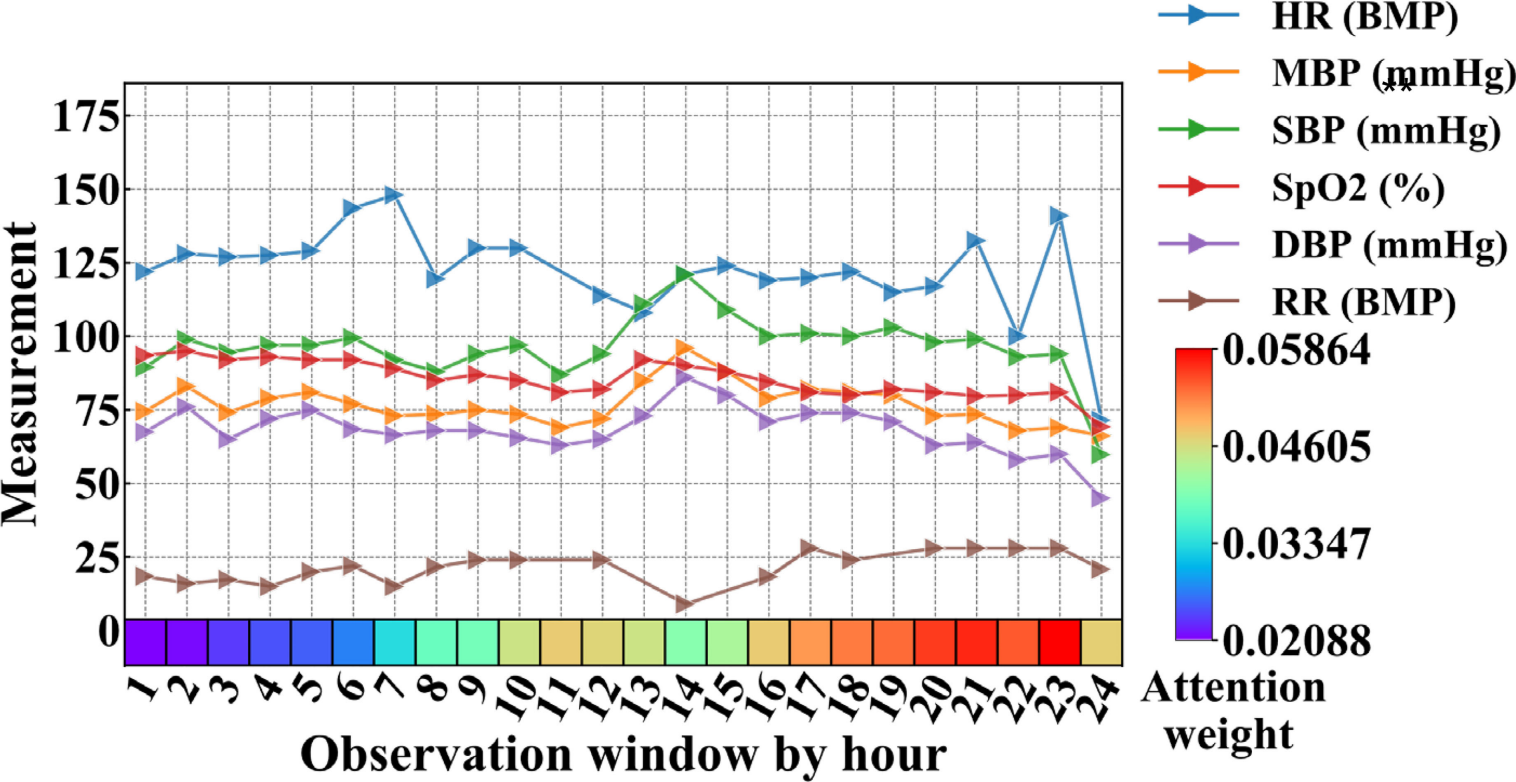
Clinical features and attention weights of one patient in the 24-hour observation window who passed away after 12 hours (prediction window, not shown in figure) from MIMIC-III. BMP, beats per minute; DBP, diastolic blood pressure; HR, heart rate; MBP, mean blood pressure; RR, respiratory rate; SBP, systolic blood pressure.

Since the deep learning model Bi-LSTM_Attn included two attention layers, we used an example patient to visualize the attention weights of temporal clinical features from the best predictive windows (24-hour observation window and 12-hour prediction window) in **Figure 9**. For non-temporal drug prescription features, the top 10 most important features were ranked by average attention weights and shown in **Supplementary Table S6**.

**Figure 9** showed the average vital signs and attention weights of the patient who suffered from severe pneumonia in MIMIC-III data, with complications of acute respiratory failure, congestive heart failure, acute renal failure and other diseases, and eventually died in the ICU (ICUSTAY_ID = 268985). We observed that during the first 23 hours of the observation window, the patient’s heart rate was too high compared to the normal range (60-100 BMP), possibly due to congestive heart failure. Since the pneumonia patient suffered acute respiratory failure, the respiratory rate (RR) increased beyond the normal range (12-20 BMP), especially in the later part of the observation window. Though the vital signs fluctuated and changed over time, the attention weights showed in the bottom row of **Figure 9** increased to a relatively larger value after the 17th hour. It reached its maximum at the 23rd hour, when all the vital signs of the patients showed a sharp drop afterwards. Though it was still 12 hours before the patient passed away, the model already picked up some unusual signs at this time point.

## 5 Discussion

In this study, we collected data of both COVID-19 and other types of pneumonia and analyzed the clinical variables that may affect the severity of COVID-19 and other types of pneumonia. Though the quick detection technology of COVID-19 has been widely adopted for diagnosis, it is still useful to do a retrospective analysis on these clinical variables to better understand the disease characteristics for better prevention towards fatal outcomes.

APACHE II score system is the most commonly used comprehensive index to assess the severity of pneumonia patients in ICUs. A higher APACHE II score indicates a worse condition and a greater risk of death for the patient. Wang et al. (2020) determined that the median of APACHE II in severe patients with COVID-19 was 17 (IQR: 10-22) and its lower quartile was slightly greater than the critical value of APACHE II in **Supplementary Figure S1**, which indicated that APACHE II < 8.5 identified by our model could be a useful cutoff to classify the severity of patients with COVID-19. Studies (Narasaraju et al., 2011; Liu et al., 2016; Lefrançais et al., 2018) have shown that excessive activation of neutrophils can release neutrophil extracellular traps (NETs), and the excessive formation of NETs can lead to a series of inflammatory reactions. These reactions may cause permanent damage to the lungs as well as the cardiovascular and renal systems in COVID-19 patients. In our results, lymphocytes are the third important factor affecting the severity of patients with COVID-19. During the clinical observation, the laboratory examination results showed that the number of lymphocytes in severe patients with COVID-19 decreased significantly, and the numbers of CD3, CD8, and CD4 T cells also continued to decline, which led to the aggravation of inflammatory reactions, increased cytokine levels and seriously damaged lung function (Chen et al., 2020; Tatum et al., 2020). Neutrophil-to-lymphocyte ratio (NLR) is an effective and convenient inflammatory marker for predicting systemic inflammation (de Jager et al., 2012; Cataudella et al., 2017; Tatum et al., 2020). However, NLR can also be used to determine the severity of the disease in patients with COVID-19. Liu et al. (2020) found that the NLRs of severe patients with COVID-19 were higher than non-severe patients. Severe patients with COVID-19 had symptoms of decreased lymphopenia and increased neutropenia, which caused the increase of proinflammatory cytokine levels in patients. The cytokine storm may make the immune system to lose control and damage the host cells, leading to multiple organ failure and patient fatality (Bermejo-Martin et al., 2020; Tan et al., 2020). Therefore, clinicians can identify high-risk COVID-19 patients using NLR. In addition to the clinical indicators discussed above, other features highlighted in **Figure 6** were also found to have associations with COVID-19. Higuera-De-La-Tijera et al. (2021) showed the aspartate aminotransferase (AST) levels of severe COVID-19 patients were significantly higher than non-severe COVID-19 patients since AST is related to liver function. Additionally, Bastug et al. (2020) identified total protein and AST as important clinical indicators of COVID-19 patients admitted to ICUs. Ou et al. (2020) found that the white blood cell counts (WBCs) and procalcitonin in severe COVID-19 patients increased significantly, indicating their clinical predictability of COVID-19 severity. The increase of blood urea nitrogen (BUN) can lead to renal function injury (STARK, 1980), which may deteriorate the severity of COVID-19 (Kang et al., 2021). Lippi and Plebani (2020) reported the procalcitonin levels of severe COVID-19 patients were twice as much as non-severe COVID-19 patients, and Oblokulov et al. (2021) found this indicator helpful to come up with the treatment plan. Zhang et al. (2020) showed that the prothrombin time (PT) and thrombin time (TT) of severe COVID-19 patients were significantly higher than those of non-severe patients, as the coagulation function were affected in most severe COVID-19 patients.

Among the top 10 most important drugs for pneumonia, ipratropium bromide, aspirin and linezolid have treatment effects. Ipratropium bromide is a safe and effective drug in the treatment of chronic obstructive pulmonary disease (COPD) (Taylor et al., 2001). When ipratropium bromide was used in the treatment of acute exacerbation of COPD, blood oxygenation did not decrease initially (Ogale et al., 2010), which helped to improve lung function and reduce the risk of pneumonia. Aspirin has antiplatelet and anti-inflammatory effects, which can reduce the risk of cardiovascular disease (Gasparyan et al., 2008). A large primary care institution in the UK has shown that aspirin can prevent pneumonia patients from cardiovascular disease, thus improving the survival rate (Hamilton et al., 2021). Meanwhile, aspirin and macrolide drugs have a synergistic effect. For ICU patients with severe pneumonia, taking aspirin and macrolide drugs at the same time can produce different complementary effects and moderate the inflammatory reaction caused by pneumonia (Falcone et al., 2019). The clinical and microbial experiments of Wunderink et al. (2012) showed that linezolid was more effective than vancomycin in the treatment of pneumonia. On the contrary, fluticasone propionate can aggravate the condition of pneumonia. Fluticasone propionate is a kind of inhaled corticosteroids (ICS) for the treatment of chronic obstructive pulmonary disease (COPD). However, it may cause bacterial infection of the respiratory system due to poor dissolution and fluid lining in the airway and increase the risk of pneumonia (Janson et al., 2017).

COVID-19 is an acute respiratory infectious disease caused by viral infection (Fauci et al., 2020), while a regular pneumonia is a respiratory infectious disease caused by bacterial or viral infection (Bartlett and Mundy, 1995). They may have different pathogens and treatment strategies. Despite the differences, there are similarities among the top contributing clinical features of the two. It is observed that lymphocytes are the third most important feature to predict COVID-19 (**Figure 6**) as well as the top 10th most important features for pneumonia (**Figure 8**). It was reported that both COVID-19 and regular pneumonia patients may experience inflammatory reactions caused by infection and result in lymphopenia (Yamasaki et al., 2020; Zhao et al., 2020). In terms of treatment, COVID-19 is mainly treated with antiviral symptomatic support approaches (Kim et al., 2020), while other types of pneumonia were usually treated with sensitive antibiotics according to the pathogen (Lutfiyya et al., 2006). A few drugs may be useful to treat both conditions. Aspirin, for example, can reduce inflammation of COVID-19 and other pneumonia by inhibiting the synthesis of prostaglandins or other substances that may cause inflammatory reactions (Hamilton et al., 2021). Among the top 10 important features of both datasets shown in **Figure 6** and **Figure 8**, lymphocytes and blood urea nitrogen (BUN) are shared indicators to predict the patient survival in MIMIC-III as well as the severity of COVID-19. For the COVID-19 data, except APACHE II, the other nine are laboratory measurements, while for the MIMIC-III data, there are six laboratory measurements and four drug treatment features (norepinephrine, vasopressin, morphine and midazolam). While the laboratory measurements are very important clinical features to predict disease outcomes in both cases, compared to COVID-19 data, the drug treatment features for MIMIC-III pneumonia patients gained more importance and may be effective in alleviating the condition of pneumonia. Since COVID-19 is a relatively new disease and drug treatments are still in active development, and the severity levels of COVID-19 and pneumonia data are different, it makes sense that more drug features were found in MIMIC-III pneumonia data. The understanding of both the similarities and differences of COVID-19 and other pneumonia may help us to monitor the critical clinical variables, make effective treatment plans and minimize fatalities. In the future work, we hope to extend the models to other disease areas and aid doctors to better understand diseases and make timely and effective clinical decisions.

The current research has a few limitations. The COVID-19 data from Wuhan, Hubei Province has a small sample size, and they only represent a very local patient pool and may not be applicable for patient groups in other regions or other COVID-19 variants. Similar limitation may also exist in the MIMIC-III and eICU database as their data are mainly from the United States. Thus, the conclusions of this study need to be tested in other large databases, cohorts and broader regions, to better identify the similarity and differences between COVID-19 and other types of pneumonia and the clinical indicators associated with them.

## 6 Conclusion

In this study, we collected pneumonia data from three sources, the COVID-19 clinical records, eICU and MIMIC-III. Statistical analysis was carried out to analyze the distributions and differences of clinical variables based on disease severeness. We developed multiple machine learning models to predict the severity of COVID-19 patients and the mortality of patients with other types of pneumonia in ICUs. The models were evaluated and compared by multiple metrics and different settings. We further analyzed the important features and interpreted the models. For the pneumonia patients, we found the same optimal time window to predict the death of ICU patients from both MIMIC-III and eICU (24-hour observation window and 12-hour prediction window). In addition, we further introduced deep learning models with two levels of attention mechanisms to visualize and interpret the temporal clinical variables *via* attention weights for pneumonia. It is observed while differences remain between COVID-19 and other pneumonia, lymphocytes are important biomarkers in both cases. This study provided a comprehensive analysis and comparison between COVID-19 and other types of pneumonia, which may have implications to aid clinicians for better disease understanding and clinical decision making.

## Data Availability Statement

The data analyzed in this study is subject to the following licenses/restrictions: Only credentialed users who sign the specified DUA can access the files. Requests to access these datasets should be directed to MIMIC-III (https://physionet.org/content/mimiciii/1.4/) and eICU (https://physionet.org/content/eicu-crd/2.0/).

## Ethics Statement

The studies involving human participants were reviewed and approved by Institutional Review Boards of Beth Israel Deaconess Medical Center (Boston, MA) and the Massachusetts Institute of Technology (Cambridge, MA), Ethics Committee of Fujian Medical University Union Hospital. The patients/participants provided their written informed consent to participate in this study. Written informed consent was obtained from the individual(s) for the publication of any potentially identifiable images or data included in this article.

## Author Contributions

Concept and design, YiZ, JW, RZ and CC. Procedures, YuZ and CC. Writing of article, YuZ, RZ, ZW, and HL. All authors have read and agreed to the published version of the manuscript.

## Funding

This work was supported by National Natural Science Foundation of China (81971837), Natural Science Foundation of Fujian Province, China (2020J05109), and Funds of Scientific Research-Support Project, Fujian Provincial Department of Finance (2021XH018).

## Conflict of Interest

Author HL was employed by the company MetaNovas Biotech Inc. at present. Related work in this study was done in Fuzhou University.

The remaining authors declare that the research was conducted in the absence of any commercial or financial relationships that could be construed as a potential conflict of interest.

## Publisher’s Note

All claims expressed in this article are solely those of the authors and do not necessarily represent those of their affiliated organizations, or those of the publisher, the editors and the reviewers. Any product that may be evaluated in this article, or claim that may be made by its manufacturer, is not guaranteed or endorsed by the publisher.
