# Supplementary material for: Statistical Analysis and Machine Learning Prediction of Disease Outcomes for COVID-19 and Pneumonia Patients
Source: Front Cell Infect Microbiol. 2022 Apr 19;12:838749. doi: 10.3389/fcimb.2022.838749 (PMC9063041; doi:10.3389/fcimb.2022.838749)
Supplement: Supplementary file 2 [file DataSheet_2.docx]

Supplementary Material

# Evaluation critera

The area under the receiver operating characteristic curve (AUROC) and area under the precision-recall curve (AUPRC) were calculated based on confusion matrices as follows.

|  |  | **Predicted** | |  |
| --- | --- | --- | --- | --- |
|  |  | **Positive (1)** | **Negative (0)** | **Totals** |
| **Actual** | **Positive (1)** | TP | FN | TP+FN |
|  | **Negative (0)** | FP | TN | FP+TN |
|  | **Totals** | TP+FN | FN+TN | TP+FN+FP+TN |

In the above confusion matrix, the following metrics can be calculated.

TP (True Positive): A sample is predicted to be positive, and its label is positive.

TN (True Negative): A sample is predicted to be negative, and its label is negative.

FP (False Positive): A sample is predicted to be positive, and its label is negative.

FN (False Negative): A sample is predicted to be negative, and its label is positive.

$$Accuracy = \frac{TP+TN}{TP+TN+FN+FP}$$

$$TPR = Recall = sensitivity = \frac{TP}{TP+FN}$$

$$Specificity = \frac{TN}{TN+FP}$$

$$FPR = 1 - Specificity = \frac{FP}{TN+FP}$$

$$Precision= \frac{TP}{TP+FP}$$

AUROC (area under the receiver operating characteristic curve): The curve drawn with false positive rate (FPR) as the X axis and true positive rate (TPR) as the Y axis is an ROC curve, and the area under the curve is AUROC. AUROC is used to measure the quality of the classification model, and the impact caused by the selection of threshold can be ignored. When AUROC is close to 1.0, the model is shown to have best prediction performance; When it is equal to 0.5, the performance is considered random.

AUPRC (area under the precision-recall curve): The precision-recall (PR) curve is drawn with recall as the X axis and precision as the Y axis. AUPRC is calculated as the area under the PR curve. The PR curve shows the trade-off between accuracy and recall across different decision thresholdss.

# Supplementary Figures

**Supplementary Figure S1.** The decision rule of key features in the XGBoost model from the COVID-19 data. APACHE II: acute physiology and chronic health evaluation II.

**
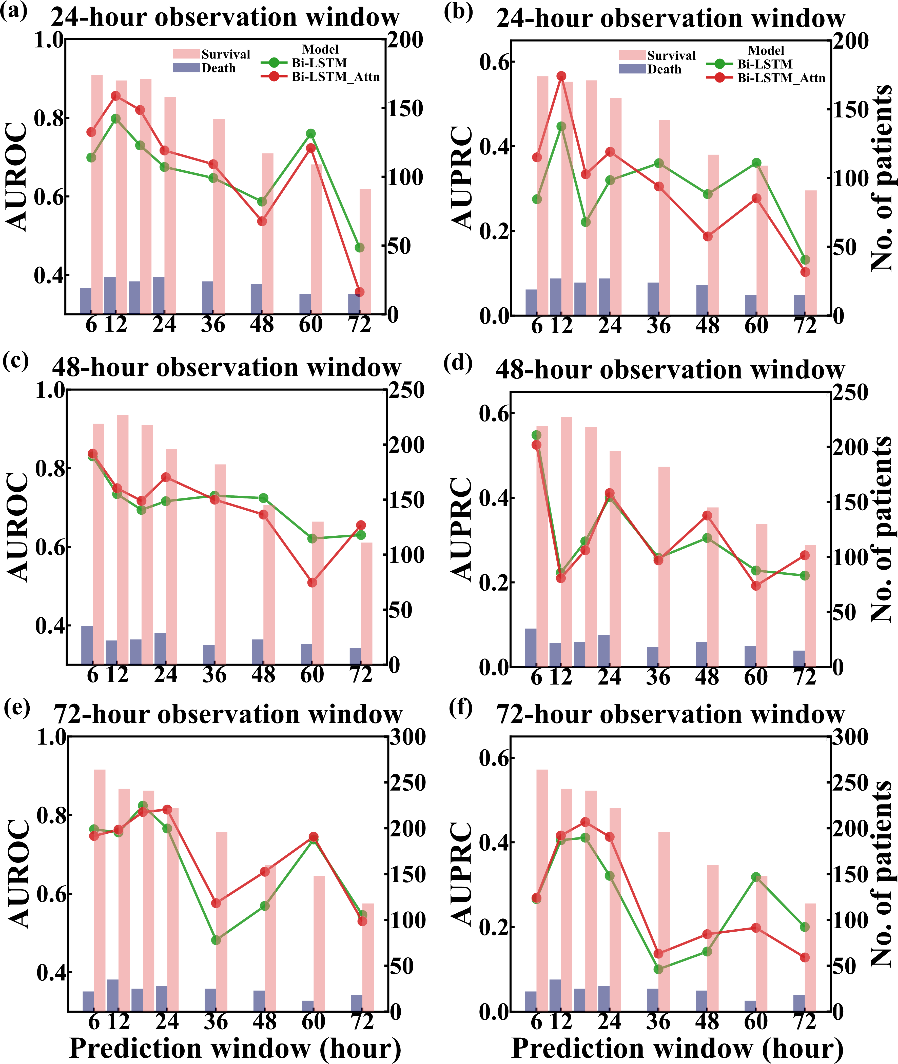
**

**Supplementary Figure S2.** Performance of our proposed model (Bi-LSTM and Bi-LSTM_Attn) to predict pneumonia patient survival using different prediction and observation windows based on the test set of eICU. The left column showed AUROC values and the right column showed AUPRC values. (a) and (b) are AUROC and AUPRC of different models under different 24-hour observation windows and different prediction windows, respectively. (c) and (d) are AUROC and AUPRC of different models under different 48-hour observation windows and different prediction windows, respectively. (e) and (f) are AUROC and AUPRC of different models under different 72-hour observation windows and different prediction windows, respectively.
